# The Prevalence of Anxiety Among University Students in the United Arab Emirates Following the COVID-19 Lockdown

**DOI:** 10.7759/cureus.56259

**Published:** 2024-03-16

**Authors:** Marwan Faris, May M Macky, Abdelrahman H Badran, Mariam Saif, Mohga Yasser, Eithar Ibrahim, Amal Hussein

**Affiliations:** 1 College of Medicine, University of Sharjah, Sharjah, ARE; 2 Department of Family and Community Medicine, University of Sharjah, Sharjah, ARE

**Keywords:** covid-19, covid-19 lockdown, covid-19 anxiety, mental health, student performance

## Abstract

Introduction: The COVID-19 pandemic, with its consequential lifestyle changes, is anticipated to contribute to increased anxiety levels, particularly among university students who already contend with significant academic stress. We aim to assess the prevalence of anxiety among university students in the United Arab Emirates (UAE) following the COVID-19 lockdown period.

Methods: We conducted a descriptive cross-sectional study among students enrolled in UAE universities. A self-administered questionnaire was utilized to gather demographic data, assess anxiety levels using the generalized anxiety disorder-7 scale, explore potential factors associated with heightened anxiety, investigate the impact of increased anxiety on academic performance, and identify coping mechanisms employed post-lockdown.

Results: Of the 369 participating students, anxiety levels were minimal in 87 (23.6%), mild in 163 (44.2%), and moderate to severe in 119 (32.2%) subjects. Moreover, increased anxiety levels were significantly correlated with poor/fair sleep quality (p=0.002). Importantly, students with moderate to severe anxiety levels exhibited poorer performance in exams and assignments (p=0.001) and encountered difficulties in maintaining focus on studies (p<0.001). The predominant coping mechanisms employed by students included self-distraction, prayer, and maintaining a positive attitude.

Conclusion: The majority of students in our study experienced mild to severe levels of anxiety following the COVID-19 lockdown period. We hope that our findings will prompt university and government officials to implement effective screening and preventive strategies to adequately support university students in future public health crises.

## Introduction

The mental well-being of university students has become a subject of increasing concern due to various stressors such as academic pressure, career uncertainties, financial challenges, and inadequate social support [1]. The emergence of the COVID-19 pandemic further exacerbated these concerns as many countries implemented lockdown measures, altering lifestyles and reducing social interactions, potentially impacting mental health. Remote learning, necessitated by lockdowns, added another layer of psychological distress for students worldwide.

A study conducted in 2018 in Hong Kong aimed to assess the prevalence of anxiety and depression among 1119 undergraduate students. The patient health questionnaire-9 and generalized anxiety disorder-7 (GAD-7) scales were utilized to screen for depression and anxiety, respectively. The findings revealed that a significant proportion of respondents reported experiencing mild to severe depressive symptoms (68.5%) and mild to severe anxiety symptoms (54.4%). These results underscore the considerable prevalence of anxiety and depression among university students [1].

Following the COVID-19 outbreak, a 2020 study conducted in China examined the impact of lockdown measures on mental health among adults aged 18 and above. The study utilized the self-rating anxiety scale and the self-rating depression scale to assess anxiety and depression, respectively. Participants were categorized into affected and unaffected groups based on whether they or their family members, colleagues, classmates, or neighbors had been quarantined. Out of the 1593 participants included in the study, a notable disparity in the prevalence of anxiety and depression was observed, where the prevalence of anxiety and depression in the group affected by quarantine was 12.9% and 22.4%, respectively, compared to 6.7% and 11.9% in the unaffected group. These findings suggest an association between quarantine measures and increased levels of depression and anxiety [2].

Elevated anxiety levels have been linked to impaired academic performance and significant functional impairment across various life domains, including diminished social relationships, poorer well-being, and decreased satisfaction with life [3-4]. Despite this, limited research has explored the mental health implications of lockdown measures among university students in the UAE population. Therefore, this study aims to assess anxiety levels among United Arab Emirates (UAE) university students and identify associated factors.

## Materials and methods

Study design and participant information

This cross-sectional study was conducted at the College of Medicine, University of Sharjah, Sharjah, United Arab Emirates (UAE). The participants of the study were students enrolled in universities across the UAE. The duration of the study was one month, and data was collected from February to March 2021. The inclusion criteria in this study were any Arabic or English-speaking student enrolled in any university in the UAE. There were no exclusion criteria in the study.

Sample size and sampling method

Volunteer sampling was used to collect data from English and/or Arabic-speaking university students studying at universities in the UAE. Based on a sampling error of 5% and 50% prevalence (p), by using n=4p(1-p)/SE^2, and accounting for a 10% nonresponse rate, our sample size was 360. Prevalence was used at 50% because we could not find any previous publication with the same aim, target population, and geographic location at the time of data collection.

Ethical approval

Ethical approval was obtained from the University of Sharjah Research Ethics Committee (approval number REC-21-02-12-01-S) and other required authorities before conducting the study. Participation in the study was entirely voluntary, with confidentiality assured and consent obtained from all participants.

Data collection instrument

A piloted, self-administered, structured online questionnaire was developed. Attached to the questionnaire were a patient information sheet and consent form, which were used to gather consent from the study participants. The questionnaire was available in Arabic and English languages. It consisted of 24 questions and was divided into five sections: demographics, the GAD-7 scale, associated factors, academic-related effects, and coping mechanisms. The GAD-7 scale is a seven-item instrument that is used to measure the severity of GAD. Scores ranging between 0 and 4 were categorized as "minimal anxiety," scores between 5 and 9 were classified as "mild anxiety," scores between 10 and 14 were labeled as "moderate anxiety," and scores between 15 and 21 were designated as "severe anxiety" (Table 1) [5].

**Table 1:** The GAD-7 questionnaire GAD-7: generalized anxiety disorder-7

	Not at all	Several days	More than half the days	Nearly every day
Little interest or pleasure in doing things	0	1	2	3
Feeling down, depressed, or hopeless	0	1	2	3
Trouble falling or staying asleep, or sleeping too much	0	1	2	3
Feeling tired or having little energy	0	1	2	3
Poor appetite or overeating	0	1	2	3
Feeling bad about yourself or that you are a failure or have let yourself or your family down	0	1	2	3
Trouble concentrating on things, such as reading the newspaper or watching television	0	1	2	3
Moving or speaking so slowly that other people could have noticed or the opposite, being so fidgety or restless that you have been moving around a lot more than usual	0	1	2	3
Thoughts that you would be better off dead or hurting yourself in some way	0	1	2	3

Statistical analysis

Data analysis was performed using SPSS Statistics, version 26.0 (IBM Corp., Released 2019; IBM SPSS Statistics for Windows, Version 26.0; Armonk, NY: IBM Corp.). Frequencies and percentages were used to report categorical data. Given the categorical nature of the collected data, the Chi-square test was employed to analyze the data and evaluate the independence or association between anxiety levels and various variables. A p-value of <0.05 was considered a statistically significant difference.

## Results

A total of 369 students were enrolled. The study cohort predominantly consisted of female participants, with the majority pursuing their studies at the University of Sharjah. Table 2 shows the demographic characteristics of the participants in our study.

**Table 2:** Demographic characteristics of participants (n=369)

Variables	Category	n (%)
Age group (years)	17-20	250 (67.8%)
	21+	119 (32.2%)
Gender	Female	304 (82.4%)
	Male	60 (16.3%)
	Prefer not to say	5 (1.4%)
Field of study	Nonmedical	207 (56.1%)
	Medical	162 (43.9%)
University	University of Sharjah	218 (59.1%)
	Higher Colleges of Technology	48 (13.0%)
	Zayed University	34 (9.2%)
	Ajman University	26 (7%)
	American University of Sharjah	14 (3.8%)
	Others	29 (7.9%)
Marital status	Unmarried	353 (95.7%)
	Married	16 (4.3%)
Working status	Unemployed	352 (95.4%)
	Employed	17 (4.6%)

**Figure 1:**
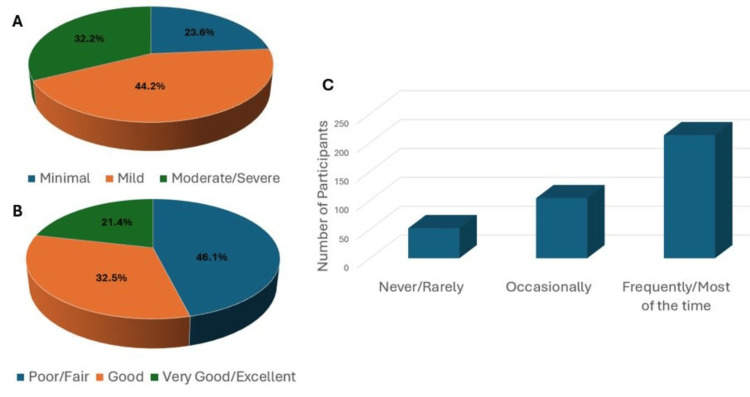
Participant characteristics (A): Pie chart demonstrating scores from the 7-item GAD-7 questionnaire showing the levels of anxiety among medical students during COVID-19. (B): Pie chart demonstrating the quality of sleep during the COVID-19 lockdown as reported by the participants. (C): Bar chart demonstrating the frequency of difficulty concentrating on studies during lockdown as reported by the participants. GAD-7: generalized anxiety disorder-7

Out of the 369 study participants, 87 (23.6%) experienced minimal levels of anxiety, while 162 (44.2%) reported mild levels of anxiety, and 119 (32.2%) suffered from moderate to severe levels of anxiety (Figure 1A). Additionally, 170 (46.1%) reported poor quality of sleep (Figure 1B), and 213 (57.7%) reported frequent difficulty concentrating on studies (Figure 1C).

Regarding the factors associated with anxiety (Table 3), a significant association between sleep quality and anxiety levels was observed. Specifically, 41.2% (n=70) of students reporting poor/fair sleep quality (n=170) exhibited moderate/severe anxiety, whereas 31.6% (n=25) of those reporting very good/excellent sleep quality (n=79) displayed minimal anxiety levels (p=0.002). When asked about the impact of their lockdown on academic performance, 58.8% (n=70) of students with moderate/severe anxiety (n=119) reported moderate/major negative effects on exam and assignment performance, compared to 33.3% (n=29) of those with minimal anxiety (n=87) (p=0.001). Additionally, 70.6% (n=84) of students with moderate/severe anxiety (n=119) reported experiencing a moderate amount/great deal of difficulty concentrating on their studies, while only 36.8% (n=32) of those with minimal anxiety (n=87) reported facing such challenges (p<0.001). We found that 48.2% (n=178) of the participants in our study were moderately/extremely concerned about contracting a COVID-19 infection during this period, compared to 25.7% (n=95) and 26.0% (n=96) of participants who were somewhat concerned and slightly/not at all concerned, respectively. There was no significant association between the level of concern about contracting a COVID-19 infection and the level of anxiety among the participants. Among the study participants, 18.4% (n=68) reported job loss within their families due to the pandemic and subsequent restrictions; 42.0% (n=155) faced financial difficulties during this time; 53.4% (n=197) reported experiencing weight gain; and 50.4% (n=186) reported rarely or never exercising at home. Despite these findings, no significant associations were observed between anxiety levels and variables such as gender, field of study, exposure to COVID-19-related news, financial status, employment status, job loss within the family, weight gain, or activity level among the participants.

**Table 3:** Factors associated with anxiety

Variable	Category	Level of anxiety			p-value
		Minimal	Mild	Moderate-severe	
Age group	17-20	58 (66.7%)	106 (65.0%)	86 (72.3%)	0.425
	21+	29 (33.3%)	57 (35.0%)	33 (27.7%)	
Gender	Male	15 (17.2%)	24 (14.7%)	21 (17.6%)	0.727
	Female	72 (82.8%)	136 (83.4%)	96 (80.7%)	
	Prefer not to say	0 (0.0%)	3 (1.8%)	2 (1.7%)	
Field of study	Nonmedical	44 (50.6%)	93 (57.1%)	70 (58.8%)	0.473
	Medical	43 (49.4%)	70 (42.9%)	49 (41.2%)	
Marital status	Single	82 (94.3%)	157 (96.3%)	114 (95.8%)	0.744
	Married	5 (5.7%)	6 (3.7%)	5 (4.2%)	
Working status	Employed	5 (5.7%)	6 (3.7%)	6 (5.0%)	0.731
	Unemployed	82 (94.3%)	157 (96.3%)	113 (95.0%)	
Sleep quality	Poor/fair	28 (32.2%)	72 (44.2%)	70 (58.8%)	0.002
	Good	34 (39.1%)	60 (36.8%)	26 (21.8%)	
	Very good/excellent	25 (28.7%)	31 (19.0%)	23 (19.3%)	
Impact on exam performance	Neutral	17 (19.5%)	41 (25.2%)	22 (18.5%)	0.001
	Minor impact	41 (47.1%)	55 (33.7%)	27 (22.7%)	
	Moderate/major impact	29 (33.3%)	67 (41.1%)	70 (58.8%)	
Difficulty concentrating	Never/rarely	19 (21.8%)	22 (13.5%)	11 (9.2%)	<0.001
	Occasionally	36 (41.4%)	44 (27.0%)	24 (20.2%)	
	Frequently/most of the time	32 (36.8%)	97 (59.5%)	84 (70.6%)	
Concern about contracting COVID-19 infection	Not at all/slightly concerned	23 (26.4%)	43 (26.4%)	30 (25.2%)	0.220
	Somewhat concerned	22 (25.3%)	50 (30.7%)	23 (19.3%)	
	Moderately/extremely concerned	42 (48.3%)	70 (42.9%)	66 (55.5%)	
Reading to COVID-19 related news	Never/rarely	31 (35.6%)	67 (41.1%)	50 (42.0%)	0.888
	Occasionally	16 (18.4%)	29 (17.8%)	22 (18.5%)	
	Frequently/most of the time	40 (46.0%)	67 (41.1%)	47 (39.5%)	
Financial difficulties	Yes	35 (40.2%)	67 (41.1%)	53 (44.5%)	0.787
	No	52 (59.8%)	96 (58.9%)	66 (55.5%)	
Job loss within the family	Yes	12 (13.8%)	35 (21.5%)	21 (17.6%)	0.317
	No	75 (86.2%)	128 (78.5%)	98 (82.4%)	
Exercise frequency	Never/rarely	43 (49.4%)	74 (45.4%)	69 (58.0%)	0.058
	Sometimes	22 (25.3%)	61 (37.4%)	33 (27.7%)	
	Frequently/most of the time	22 (25.3%)	28 (17.2%)	17 (14.3%)	
Weight gain	Yes	46 (52.9%)	81 (49.7%)	70 (58.8%)	0.314
	No	41 (47.1%)	82 (50.3%)	49 (41.2%)	

Regarding coping mechanisms, the most prevalent strategy among participants (n=280, 75.9%) was self-distraction, followed by praying (n=199, 53.9%), maintaining a positive attitude (n=184, 49.9%), learning new skills (n=135, 36.6%), humor (n=119, 30.1%), binge eating (n=117, 31.7%), venting (n=111, 30.1%), seeking emotional support (n=73, 19.8%), seeking advice (n=72, 19.5%), engaging in self-blame (n=69, 18.7%), and smoking (n=30, 8.1%).

## Discussion

In this study, we investigated the levels of anxiety among university students in the UAE following the COVID-19 lockdown period. Our findings revealed that a majority of students (n=162, 44.2%) reported mild levels of anxiety, while nearly a third (n=119, 32.2%) of the participating students (n=369) reported moderate to severe levels of anxiety. Poor sleep quality was found to be significantly associated with heightened anxiety levels. Furthermore, students experiencing elevated anxiety levels encountered difficulties concentrating on their studies and demonstrated poorer performance in exams and assignments.

A prior study utilized the GAD-7 scale to assess anxiety levels among 404 university students enrolled in a single university in the UAE before the lockdown, revealing moderate to severe anxiety in less than a quarter (22.3%) of the cohort [6]. Our study suggests an escalation in the prevalence of moderate to severe anxiety following the implementation of COVID-19-related lockdown measures in the UAE. Similar findings associating increased anxiety during lockdown with restricted physical activity, limited social interaction, and disruptions in routine activities have been documented [7]. Islam et al. attributed the heightened levels of anxiety in university students to a blend of academic pressures, such as the challenge of meeting academic requirements, and professional uncertainties, including the prospect of unemployment and subsequent financial insecurity [5]. In contrast, our study did not reveal a significant impact of unemployment or financial insecurity on the levels of anxiety among the participants, despite nearly a fifth of the cohort reporting the loss of a job within their family and almost half reporting financial difficulties.

Our results align with a recent study in the UAE conducted around the lockdown period, which assessed the anxiety levels of 1485 university students using the GAD-7 scale and found that over half of the enrolled participants reported anxiety levels ranging from mild to severe [8]. Notably, this study reported a significant increase in anxiety levels among females compared to males, contrasting with our findings, where no significant gender difference was observed. This discrepancy may be attributed to the relatively small proportion of male participants in our study, reflecting the gender imbalance in higher education in the UAE [9].

We observed a significant association between anxiety levels and difficulties in concentration, consistent with findings from a study conducted in Saudi Arabia, where more than half of the participants reported a lack of concentration and difficulty focusing due to the pandemic's impact on mental well-being [10]. Previous literature has also described that anxiety results in increased distractibility, attentional lapses, an inability to maintain attention, and intrusive thoughts [11], all of which can negatively impact academic performance.

Previous research has underscored the prevalence of poor sleep quality among students and its positive correlation with anxiety and depression [12-13]. Marelli et al. concluded that COVID-19 lockdown measures led to increased bedtime hours, extended sleep latency, delayed wake-up times, deterioration in sleep quality, and worsening of insomnia symptoms [13]. Our findings support these associations, as evidenced by 46.1% (n=170) of respondents reporting subjectively poor/fair sleep quality and a significant positive correlation observed between poor sleep quality and heightened anxiety levels. Moreover, prior studies have elucidated the adverse impact of poor sleep quality on academic performance, as well as negative effects on health, emotional well-being, and overall quality of life [14-15].

Moreover, our results support existing evidence demonstrating a negative impact of higher anxiety levels on academic performance [3]. This relationship has been attributed to factors such as lack of energy and motivation, dissatisfaction with the university learning experience, and decreased concentration [16]. A longitudinal study among 121 medical students in Hong Kong suggested that anxiety may be caused by low academic performance rather than the opposite [17].

Limitations of our study include the potential for recall bias due to the self-administered nature of the questionnaire and the cross-sectional study design, which precludes establishing causality. Additionally, our sample predominantly consisted of females and students from the University of Sharjah, which may limit the generalizability of our findings to all UAE students. Future research employing longitudinal designs and larger sample sizes would enhance our understanding of the relationship between global pandemics, lockdown measures, and anxiety in university students.

## Conclusions

Anxiety-related disorders are widespread among university students, stemming from the numerous stressors encountered during their academic journey. Our study findings indicate that close to half of the participants experienced mild levels of anxiety, while nearly a third suffered from moderate to severe levels of anxiety. In line with our findings, several studies have highlighted a significant uptick in anxiety prevalence among this demographic amid the COVID-19 pandemic and subsequent lockdown measures. These effects have been compounded by factors such as diminished sleep quality and compromised academic performance.

Given these findings, it is encouraged for both university and governmental authorities to take proactive steps in implementing effective screening and preventive measures to support students during future public health crises. Additionally, raising awareness of the mental health challenges faced by university students and advocating for universities to offer support and promote healthy coping strategies are crucial. Such interventions are vital for safeguarding students' mental well-being and fostering resilience in the face of unprecedented challenges.
